# *Agrobacterium rhizogenes*-mediated hairy roots transformation as a tool for exploring aluminum-responsive genes function

**DOI:** 10.4155/fsoa-2018-0065

**Published:** 2019-02-08

**Authors:** Abhijit A Daspute, Xian Yunxuan, Minghua Gu, Yuriko Kobayashi, Sopan Wagh, Archana Panche, Hiroyuki Koyama

**Affiliations:** 1 Laboratory of Plant Cell Technology, Faculty of Applied Biological Sciences, Gifu University, Gifu 501–1193, Japan; 2 Institute of Bioscience & Biotechnology, Department of Biological Sciences, MGM College, Aurangabad 411-003, India; 3 State Key Laboratory for Conservation and Utilization of Subtropical Agro-bioresources, College of Agriculture, Guangxi Universities, Nanning 530-005, China

**Keywords:** *Agrobacterium rhizogenes*, *Agrobacterium tumefaciens*, aluminum, *AtALMT1*, citrate, GFP, hairy roots, malate, *STOP1*, tobacco

## Abstract

**Aim:**

To develop a useful alternative approach to evaluate the gene function in hairy roots.

**Methods:**

*Arabidopsis* and tobacco (wild-type or mutant) were a host for *Agrobacterium rhizogenes* transformation.

**Results:**

The hairy roots formation efficiency ranged from 53 to 98% in tobacco and 53 to 66% in *Arabidopsis*. Hairy and intact roots showed similar gene expression pattern in response to salt and aluminum stress. Genomic polymerase chain reaction and fluorescent images showed high rate (>80%) of co-integration of T-DNAs and uniform cell transformation without use of any antibiotic selection. Whole processes of hairy roots were completed within 1 month after the infection of *Agrobacterium*.

**Conclusion:**

Aluminum-responsive orthologous gene function could be evaluated by *NtSTOP1-*KD and *Atstop1* as a host for hairy roots transformation.

## Lay abstract

Hairy roots have been used for various purposes over the last several years. In the present study we used *Agrobacterium rhizogenes*-mediated hairy roots as an alternative approach for *Agrobacterium tumefaciens* transformation. We developed a simple, effective and reproducible hairy root protocol in tobacco and *Arabidopsis*. Developed hairy roots were compared with intact roots to characterize the gene response to aluminum, NaCl stress and cellular localization of genes. Aluminum-responsive orthologous genes function could be evaluated by using *NtSTOP1-*KD and *Atstop1* as a host for hairy roots transformation.

Recent molecular biological studies, in particular those using model plant systems such as *Arabidopsis thaliana* and rice (*Oryza sativa*), have identified various genes that regulate stress tolerance. Further studies of orthologs in crops provide valuable information for breeding of stress-tolerant varieties by molecular breeding. A number of the dehydration-responsive element binding protein (*DREB*) family, encoding transcription factors regulating drought, cold and salt tolerance, were originally identified in *A. thaliana*. Functional orthologs were identified from various major crops such as rice, soybean and from drought tolerance crop cowpea [1–3]. Several Al tolerance genes, such as the genes encoding aluminum-activated transporter1 (ALMT1) and multidrug and toxic compound extrusion1 (MATE1) of malate and citrate transporters, respectively, were identified from various crop plants [4–6]. These genes could be useful for molecular breeding in each crop species to improve stress tolerance.

Ectopic expression in different organisms is frequently used to characterize function of orthologs of crop species. Ectopic expression in oocytes or yeast is useful to study the molecular function of orthologs but *in planta* assay would have advantages to characterize its physiological functions. For example, in planta assay enables testing of orthologs if they regulate tolerance at a tissue or plant level. In fact, several transcription factors, including *DREB, NAC* and *bZIP*, were characterized by gene overexpression.

*Agrobacterium*-mediated transformation has been frequently used in planta assay. *Agrobacterium* species mostly are pathogenic, generating tumor and hairy roots in plant by integration of T-DNA containing pathogenic genes encoding phytohormone and opine synthesis enzymes [7]. *Agrobacterium tumefacience* has been used in planta assays after the establishment of nonpathogenic and small-sized plasmids, the so called mini-Ti (tumor-inducing) plasmid, and nonpathogenic strains lacking native T-DNA [8]. Although the bacterium infects mostly dicots [7] in the natural environment, it can be used for transformation in various plants including monocots in laboratory conditions [9]. This was established by modifications such as very high density infection (rice early protocol), and addition of the chemical activator (e.g, acetosyringone) of virulence reactions [10]. An efficient and reliable transformation system was developed in the medically important planta *Papaver bracteatum* and *Nepeta pogonosperma* through optimization of several factors such as bacterial strain and nutrient composition that affect the rate of *A. rhizogenes*-mediated transformation and growth rate of hairy root [11,12]. In addition, an *A. tumefacience* method has been adapted in a transient assay that enables study of intracellular localization of proteins [13]. However, there remain technical limitations for evaluating gene function. For example, it takes time to generate stable transgenic seed progenies. Also, it sometimes requires using uncommon antibiotic selection markers if subsequently transformed to transgenic plant materials such as T-DNA insertion mutants of *Arabidopsis*, which are used frequently as host of *in planta* complementation assays.

Mostly, the native strain of *A. rhizogenes* carrying pathogenicity (i.e., inducing hairy roots) could be used for evaluating function of genes *in planta*, if the phenotype can be observed in hairy roots. In fact, it has been used for studying synthesis of secondary metabolites in medicinal plants [14–17], while it may also be applied to study functional genes that regulate tolerance of roots to rhizotoxic ions. Although the concept has been applied for evaluating Al tolerance genes (i.e., gene-encoding MATE-citrate transporter of eucalyptus using wild-type tobacco [18]), it has not been evaluated whether it can apply to the complementation assays using transgenic mutant lines as host plant. By the complementation assay, it is better if the ortholog or other genes could be introduced without using any antibiotic selection marker, because the transgenic host plants usually carry marker genes for antibiotics. In the present study, we analyzed efficiency of transformation of target genes in the hairy roots, which were developed using transgenic host background. We also compared stress response of hairy and intact roots. Our analysis identified that hairy roots would be useful to study functions of transgenes that are responsible for regulation of stress tolerance in plant.

## Materials & methods

### Plant materials

*Arabidopsis* (Columbia [Col-0] and the *stop1* mutant [Col-0 background; Iuchi *et al*., 2007]); tobacco (*Nicotiana tabacum*) (wild-type [cv. Xanthi] and homozygous T_3_ generation of *NtSTOP1*-KD [*NtSTOP1*-KD; RNA interference line of *NtSTOP1*], which were used in the previous study [19]), were used as the host of transformation in the present study. The *NtSTOP1*-KD was kanamycin (kan)- and hygromycin-resistant due to its caring of the selection marker of T-DNA. A hyper virulence strain ATCC15834 (Import permit No. 26N-406, MAFF, Japan) was used for *A. rhizogenes*-mediated transformation.

### Plasmid DNA constructs & development of hairy roots

The overexpression constructs ([35S*:AtSTOP1:sGFP*] [synthetic green florescence protein] and *35S:CcMATE1:sGFP*]) were similar from the previous studies [19,20]. Briefly, the cDNA sequence of *AtSTOP1* and *CcMATE1* were attached to the 5′ end of the open reading frame of the *sGFP* gene connected to the nopaline synthase terminator by overlapping extension polymerase chain reaction (PCR) [21]. The amplicon of the respective gene was digested with *SfiI* and then introduced into the transfer DNA region of pBE2113 [22], which contains a kan resistance gene as the selection marker. Similarly, the cDNAs of *GUS* and *sGFP* were digested with Sfi1 and then introduced into T-DNA region of pBE2113 under the control of cauliflower mosaic virus 35S promoter. These constructs were transformed into *A. rhizogenes* by electroporation and used for transgenic hairy roots (*A. rhizogenes* with Mini Ti plasmid) transformation or wild-type hairy roots (only *A. rhizogenes* infection) in *Arabidopsis* as well as tobacco (Supplementary Information). Similarly, the 35S*:AtSTOP1:sGFP* construct was used for *A. tumefaciens*-based plant transformation using the floral-dip method [23].

### Culture condition & RNA extraction

*Arabidopsis* and tobacco seedlings were grown hydroponically according to the method described [19]; the culture solutions were renewed after every 2 days. The 1-month old transgenic hairy roots were precultured for 3 days in 1/50 MGRL (Modified Growth Regulators)  solution with 1% sucrose.* Arabidopsis* (10 days old; 10 μM Al, 50 mM NaCl; pH 5.0 for transcript and 5 days old; 10 μM Al; 1% sucrose; pH 5.0 for malate; as described by [19]), tobacco (14 days old; 30 μM Al, pH 5.0 for transcript and 7 days old 30 μM Al; 1% sucrose; pH 5.0 for citrate; as described by [24]) plant and 3 days precultured (both *Arabidopsis* and tobacco) hairy roots were used. RNA isolation and cDNA synthesis were performed as described by [25].

### Collection of root exudates, & quantification of citrate & malate

Citrate and malate were quantified by the enzyme reaction (citrate lyase [EC 4.1.3.8] for citrate, malate dehydrogenase [EC 1.1.1.82] for malate)-coupled NADH/NAD+ cycling method developed by [26] with minor modifications as described by [27].

### GUS staining & microscopic imaging

The GUS staining was performed as described by [28]. Cellular localization and uniform transformation of 35S*:AtSTOP1:sGFP* and *35S:CcMATE1:sGFP* were analyzed based on sGFP fluorescence. The samples were observed and photographed under an Olympus BX51 microscope 9 (Olympus, Tokyo, Japan) equipped with Olympus DP70 Camera System (Olympus) and fluorescence microscope (AXIO imager system, Carl-Zeiss-Japan, Tokyo, Japan). The transgenic hairy roots were examined for the integration and co-integration of the transformed gene by genomic PCR.

## Results

### Establishment of efficient hairy roots

**Figure 1.**
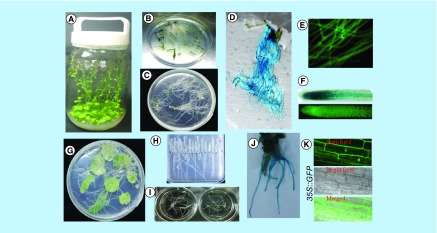
**Development of *Agrobacterium rhizogenes-*mediated transformation into *Arabidopsis* and tobacco hairy roots.** **(A)** *Arabidopsis thaliana* plants growing in containers. **(B)** *Arabidopsis* explants used for *Agrobacterium rhizogenes* infection. **(C)** Transgenic hairy root formation of *Arabidopsis*. **(D, E & F)** Transgenic hairy roots expressing the CaMV 35S-driven *GUS* and *sGFP* gene. **(G)** Hairy root formation of tobacco. **(H & I)** Excised tobacco hairy roots growth on solid and liquid medium. **(J)** Transgenic hairy roots of tobacco expressing the CaMV 35S-driven *GUS* gene. **(K)** Confocal laser scanning microscopy image of CaMV 35S-driven *sGFP* localization in tobacco transgenic hairy roots. CaMV 35S: Cauliflower mosaic virus35S.

We developed hairy root induction in *Arabidopsis* and tobacco by co-cultivating the explants with *A. rhizogenes*. In the preliminary experiment we used different explants such as leaf or stem of *Arabidopsis* or leaf or tobacco for hairy root development. The preliminary experimental results showed that stem of *Arabidopsis* and leaf of tobacco produced high efficiency of hairy roots compared with other explants (data not shown). We observed induction of tobacco and *Arabidopsis* hairy roots within <10 days, and the whole process of hairy roots was completed within 1 month from the day of infection with high efficiency and frequency of transformation (Figure 1). We obtained a very high rate of hairy roots formation efficiency in tobacco that ranges from 53 to 98%, and 58 to 66% in *Arabidopsis* (Table 1). These results showed that hairy roots can be an approach for gene analysis.

**Table 1.** **Efficiency of hairy roots formation in different batch of transformation.**

**Host plant**	**Plasmid constructs**	**No. of explants used for co-cultivation**	**No. of explants induced hairy roots**	**Hairy roots induction (%)**
*Arabidopsis* (Col-0)	*35S::GUS*	53	35	66

	*35S::GFP*	49	32	65

	*AtALMT1::GUS*	40	30	75

	*35S::STOP1:GFP*	53	31	58

Tobacco (Xanthi)	*35S::GUS*	45	44	98

	*35S::GFP*	42	15	36

	*35S::CcMATE1:GFP*	56	18	32

*NtSTOP1*-KD	*35S::GUS*	37	34	92

	*35S::GFP*	34	19	56

	*35S::CcMATE1:GFP*	58	31	53

### Transcript & organic acid analysis in *A. rhizogenes*-derived hairy roots & intact roots of wild-type or stop1 mutant of *Arabidopsis*

**Figure 2.**
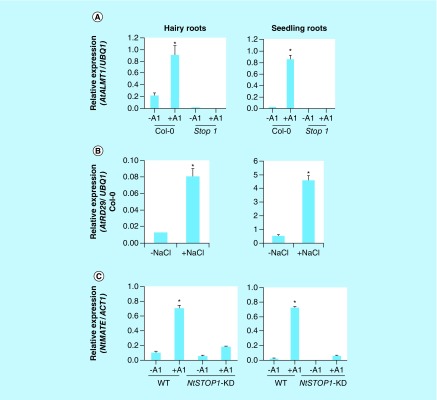
**Transcript analysis of hairy and intact roots of *Arabidopsis* and tobacco exposed to rhizotoxic stress.** Expression of *AtALMT1* **(A)**, *RD29* **(B)**, and *NtMATE* **(C)**, in wild-type hairy roots (only *Agrobacterium Rhizogenes* infection) and intact roots exposed to various rhizotoxic stress with (pH 5.0; [10 μM- : *Arabidopsis*, 30 μM- : tobacco Al, and 50 mM NaCl)] or without (pH 5.0; Al and NaCl) for 24 h was detected by RT (real-time)-polymerase chain reaction. Hairy roots were precultured for 3 days in MGRL solution containing 1% sucrose. Ubq1 and *ACT1* were used as the internal reference gene. Error bars indicate ± standard deviation (n = 3). The primers used for qRT-polymerase chain reaction were similar used from previous studies of Sawaki *et al*. and Ohayama *et al*.

To explore the molecular function and functional similarity in response to stress physiology between *A. rhizogenes*-derived hairy roots and intact roots of *Arabidopsis* or tobacco, hairy and intact roots of *Arabidopsis* were exposed to Al or NaCl stress. Hairy and intact roots of Col-0 showed Al-induced *ALMT1* and *MATE1* expression, stop1 mutant hairy roots and intact roots showed the suppression of Al-induced *ALMT1* and *MATE1* expression (Figure 2A & B). The NaCl-exposed hairy and intact roots of Col-0 showed similar expression pattern of *RD29A* gene (Figure 2B). Similarly, the *NtMATE* expression was induced by Al treatment in wild-type tobacco intact roots and suppression in *NtSTOP1*-KD intact roots. The Al responsive *NtMATE* expression pattern in hairy roots was similar with intact roots (Figure 2C). These results revealed that roots derived from *A. rhizogenes* transformation or the intact roots showed similar expression level of compared genes when exposed to various stress condition (Figure 2A, B & C). Al-responsive malate secretion is regulated by *ALMT1* in *Arabidopsis* and stop1 mutant could not excrete the malate when it is exposed to Al stress [29]. Hairy or intact roots of col-0 showed that Al-responsive malate secretion is higher than compared with control, no Al stress (Supplementary Figure 1A), whereas stop1 mutant intact and hairy roots could not excrete Al-responsive malate (Supplementary Figure 1A). On the other hand, Al-inducible citrate secretion was not observed in both hairy and intact roots of *Arabidopsis* (Supplementary Figure 1B). Hairy and intact roots of tobacco showed the highest concentration of citrate secretion with Al inducible excretion pattern (Supplementary Figure 2A); however, *NtSTOP1*-KD hairy and intact roots showed suppressed Al-inducible citrate excretion (Supplementary Figure 2A). Suppression of Al responsive citrate secretion was similar to the previous report of [24]. These results suggest that loss-of-function mutant (stop1 and *NtSTOP1*-KD) hairy roots can be used for gain-of-function analysis of genes related with Al tolerance from different plants, in particular those that are regulated by *STOP1/MATE* system.

### Microscopic imaging & cellular localization pattern

**Figure 3.**
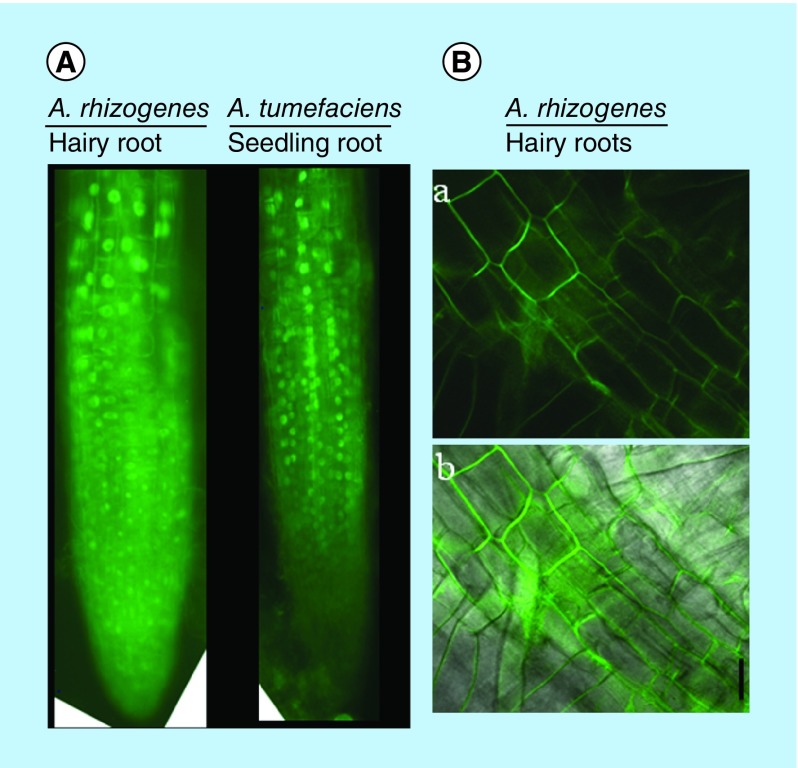
**Protein localization in *Agrobacterium rhizogenes-*transformed hairy roots and cellular transformation.** **(A)** Fluorescent microscopic image of *35S:AtSTOP1:sGFP* localization in hairy and intact roots of *Arabidopsis*. **(B)** *35S:CcMATE1:sGFP* localization in tobacco hairy roots and detection of cellular transformation base on GFP fluorescence. The confocal laser scanning microscopic image of *CcMATE1* localized in cell plasma membrane of tobacco hairy roots **(A)** (dark field) and **(B)** (merged). Uniform and single cell transformation of *CcMATE1* was detected based on GFP fluorescence. GFP: Green fluorescent protein.

In the present study we found that there were no differences in the localization pattern of *STOP1:sGFP* in *A. rhizogens*-derived hairy roots and primary roots derived from *A. tumefaciens* (Figure 3A). Similarly, the tobacco hairy roots showed *CcMATE1:sGFP* localized in the plasma membrane (Figure 3B). Also, we observed a high rate of uniform cell transformation at cellular level based on *sGFP* fluorescence (Figure 3B).

### Estimation of the ratio of co-integration of T-DNAs from Ti plasmid

**Figure 4.**
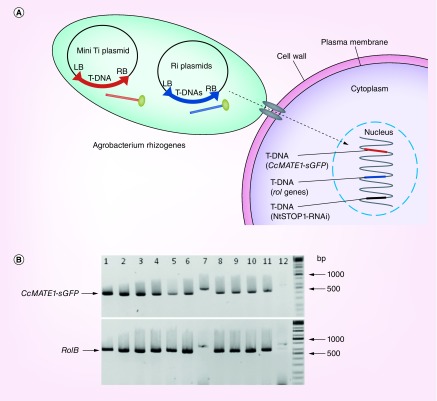
**Diagrammatic model and co-transformation efficiency of Ri and mini Ti plasmid in *Agrobacterium rhizogenes* developed tobacco hairy roots.** **(A)** Diagrammatic model illustrating co-integration of T-DNAs from mini Ti and Ri plasmid into the hairy roots of *NtSTOP1*-KD tobacco. Transgenic *Agrobacterium rhizogenes* carries two Ti plasmid; (1) T-DNA of mini Ti plasmid carries *CcMATE1-sGFP*, kanamycin as antibiotic resistance marker gene; (2) T-DNA of Ri plasmid carries *root locus* (*rol; rolA*, rolB and *rolC*) gene, responsible for opine production. The T-DNA of *NtSTOP1*-KD tobacco host plant carries sequence of STOP1-RNAi as well as kanamycin and hygromycin resistance antibiotic marker genes. **(B)** Genomic DNA was extracted from roots transformed with *CcMATE1-sGFP*, and genes were amplified using primers flanking the target sequence. The *rolB* gene present in the Ri plasmid of *A. rhizogenes* and *CcMATE1-sGFP* present in mini Ti plasmid. Presence of amplicon of hairy roots marker gene (rolB; 670 base pair) and mini Ti plasmid caring *GUS* (500 base pair) showed co-transformation. Lanes 1–11, tobacco hairy roots; lane 12, negative control (*NtSTOP1*-KD tobacco). The primer pairs of 5′-TAGCCGTGACTATAGCAAACCCCTCC-3′ and 5′-GGCTTCTTTCTTCAGGTTTACTGCAG-3′, and 5′-TACCCCGACCACATGAAGCAG-3′ and 5′- TACTTGTACAGCTCGTCCATGC-3′, were used for amplification of *RolB* and *CcMATE1-sGFP* genes, respectively. Ri: Root-inducing; Ti: Tumor-inducing.

To confirm the transformation of hairy roots, we performed genomic PCR of tobacco hairy roots (Figure 4) and reporter assays of both tobacco and *Arabidopsis* (Figure 1D & J). We examined the integration of T-DNA of Ti and Ri (root-inducing) plasmids by genomic PCR. In this analysis, hairy roots were derived with the *A. rhizogenes*-carrying *CcMATE1-sGFP* in the T-DNA of Ti plasmid. Genomic PCR of hairy root gDNA amplified the expected size of amplicons; approximately 700 base pair with *rol B* primer, and 500 base pair with *GFP* primers (Figure 4). The rate of co-integration was estimated as very high >80%. It indicated that a high rate of co-integration occurred in the hairy root generated by *A. rhizogenes*. These trends were confirmed by the GUS staining of hairy roots, which showed blue color in almost all of the hairy roots (Figure 1D & J).

## Discussion

In this study, we developed an efficient hairy root system using wild-type and mutant *Arabidopsis* or tobacco, by *A. rhizogenes-*mediated transformation, it allow us for rapid functional testing of genes (Figure 1). The *stop1* is sensitive to proton and Al rhizotoxicity [29] and suppresses the expression of Al and H tolerance in *Arabidopsis* [30]. The hairy roots of the *stop1* mutant showed suppression of Al responsive genes (Figure 2A), and were not able to secret malate (Supplementary Figure 1A). This trend of gene expression and organic acid (OA) secretion of *stop1* mutant hairy roots is very similar to intact roots of the *stop1* mutant (Supplementary Figure 1A). Moreover, the *stop1* mutant showed suppression of Al responsive *ALMT1* and malate secretion [29]. On the other hand, the salt responsive *DR29* gene revealed very similar gene expression pattern with hairy and intact roots of *Arabidopsis* (Figure 2B). Moreover, *NtSTOP1*-KD was generated by *A. tumefaciens-*mediated transformation using a vector carrying an RNAi that targeted sequences of *NtSTOP1* [24]. Hairy roots of *NtSTOP1*-KD showed suppression of *MATE, ALS3* gene and showed suppression of citrate excretion in response to Al stress. The suppression pattern of hairy roots of *NtSTOP1*-KD is very similar to that of intact roots of tobacco (Figure 2C & Supplementary Figure 2A). However, the expression patterns of the genes relating to reactive oxygen species (ROS) are different between the intact and the hairy roots, possibly because they are hairy roots [31]. Moreover, Al-tolerant cultured cells of tobacco showed Al tolerance and enhanced citrate excretion [32]. Anatomy of tomato hairy roots is similar to that of primary roots; the only difference found was that hairy roots often contain one extra cortex layer [33]. The *TaNHX2* gene could enhance salt tolerance of soybean, and the *A. rhizogenes*-mediated transformation system could be used as a complementary tool of *A. tumefaciens*-mediated transformation to rapidly investigate candidate gene function in soybean [34]. This suggests that hairy roots of mutants can be used as a background for further characterization of Al-responsive genes.

*A. tumefaciens-*mediated transient assay is a popular method to characterize gene localization. However, it needs a high level of skill to analyze results, and usually results in chimeric-transformed cell. *A. rhizogenes*-transformed hairy roots are an alternative approach to *A. tumefaciens* to study the gene localization (Figure 3A & B). *A. tumefaciens* and *A. rhizogenes*-transformed roots expressing a *GFP-GUS* transgene driven by the tomato *SHR* promoter (*SlSHR*) showed the same pattern of *GUS* and *GFP* fluorescence in the root stele of both primary and hairy roots [33]. Multiple gene transformation can be achieved by sequential transformation. Such a method is time consuming, labor intensive and use of a different antibiotic resistance gene in each step of transformation or elimination of the antibiotic resistance gene from an earlier transgenic material is important. However, in the present study we used *NtSTOP1*-KD transgenic tobacco that carries kan and hygromycin antibiotic resistance genes, as a host for hairy roots production. Besides this, mini Ti plasmid carries *CcMATE1-sGFP*, also containing kan as an antibiotic selection marker. By using *A. rhizogenes* we successfully generated transgenic tobacco hairy roots caring *CcMATE1-sGFP* without using any antibiotic selection (Figure 4). Moreover, co-transformation is an efficient approach that facilitates the delivery of more than one gene simultaneously. Co-transformation efficiency is very important in *A. rhizogenes*-mediated hairy roots transformation for generating transgenic hairy roots. Because of this, the gene of interest is present in the T-DNA region of mini Ti plasmid (transformed vector) and genes responsible for hairy roots production (i.e., *rolA, rolB, rolC* and *rolD)* were present in T-DNA of Ri plasmid. Some co-transformation strategies have been reported by using microprojectile bombardment [35]. The *CcMATE1-sGFP-*transformed *NtSTOP1*-KD tobacco hairy roots revealed very high (>80%) co-transformation efficiency by PCR analysis (Figure 4). Moreover, overexpressed GUS/GFP also showed high transformation efficiency (>95%) in both tobacco and *Arabidopsis*, using GUS/GFP fluorescence (Figure 1D & J). An average of 62% co-transformation efficiency using fluorescent markers in *Eucalyptus grandis* hairy roots by *A. rhizogenes* transformation was reported [36]. Approximately 91% of hairy root transformation efficiency was obtained using the optimum conditions of *A. rhizogenes* K599 caring 35S::*AhAREB1*-GFP [37]. A recent study showed that the hairy root induction efficiency of potato could depend on the cultivars [38]. Similarly, we found higher efficiency of hairy root infection in tobacco camper with *Arabidopsis*.

## Conclusion

We developed an efficient and simple *A. rhizogenes*-mediated hairy root development protocol in *Arabidopsis* and tobacco. The transgenic background can be used as host, without considering the antibiotic resistance gene in the transgenic background; the new gene can be efficiently introduced using *A. rhizogenes* transformation. The use of transgenic mutant background (i.e.*, NtSTOP1*-KD or stop1 mutant) as a host for *A. rhizogenes*-mediated hairy roots transformation is a prominent approach for characterizing Al-responsive orthologous genes.

## Future perspective

Hairy roots have been used for various purposes over the last several years; mostly for the production of secondary metabolite production. Recently, hairy root has been utilized as a biotechnological tool in different plant species to analyze gene function, promoters and cellular localization of proteins. Hairy root is a fast, simple and highly efficient system for utilization in biotechnology applications. However, maintenance and storage of hairy roots is still challenging and several previous reports revealed contradictions in transgene expression in intact and hairy roots. Further studies need to address issues such as storage, maintenance and effect of *Rol* genes that affect the expression of transgenes in diverse stress response.

**Summary points**

- Hairy roots have been used for various purposes over the last several years.
- We used *Agrobacterium rizogenes*-mediated hairy roots as an alternative approach for *Agrobacterium tumefaciens* transformation.
- We developed a simple, effective and reproducible hairy root protocol in tobacco and *Arabidopsis*.
- Developed hairy roots were compared with intact roots to characterize the gene response to Al and NaCl stress.
- Al and NaCl-responsive gene expression was found to be similar in hairy and intact roots of *Arabidopsis* and tobacco.
- Similarly, organic acid excretion in response to Al stress was found to be similar in intact and hairy roots of *Arabidopsis* and tobacco.
- *Atstop1* mutant and *NtSTOP1*-KD were used as a host background for hairy root developments of *Arabidopsis* and tobacco, respectively.
- Protein localization of STOP1 and CcMATE1 was found to be similar in intact and hairy roots of *Arabidopsis* and tobacco.
- Genomic polymerase chain reaction and confocal imaging analysis revealed high co-transformation efficiency of T-DNA of mini Ti and Ri plasmids without use of any selection pressure.
- *Atstop1* mutant and *NtSTOP1*-KD can be used as a host background for hairy root transformation to evaluate the Al-responsive genes from orthologous plant species.
