# Supplementary figures and images for: Agrobacterium rhizogenes-mediated hairy roots transformation as a tool for exploring aluminum-responsive genes function
Source: Future Sci OA. 2019 Feb 8;5(3):FSO364. doi: 10.4155/fsoa-2018-0065 (PMC6426172; doi:10.4155/fsoa-2018-0065)

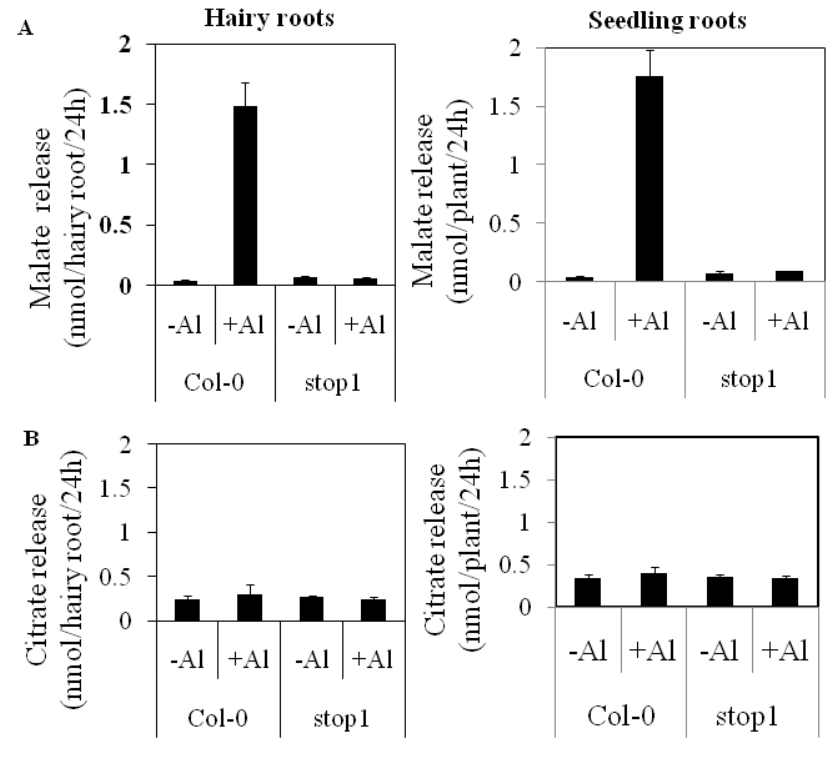

Supplement: Supplementary file 1 [file fsoa-05-364-s1.tif]

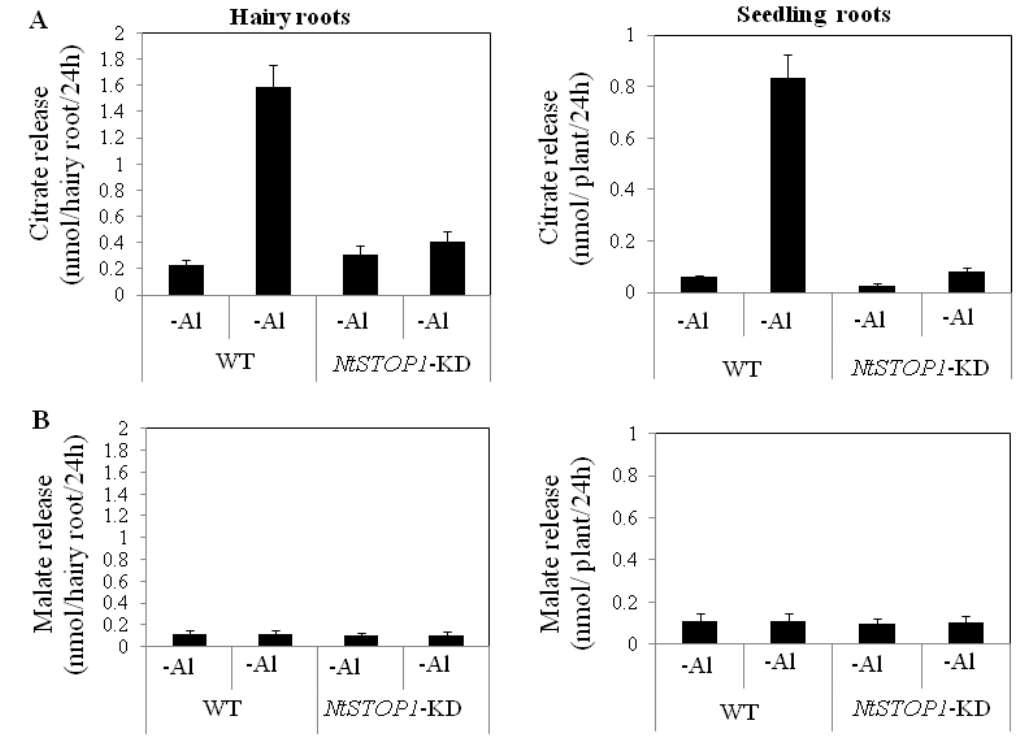

Supplement: Supplementary file 2 [file fsoa-05-364-s2.tif]
