# Supplementary material for: Agrobacterium rhizogenes-mediated hairy roots transformation as a tool for exploring aluminum-responsive genes function
Source: Future Sci OA. 2019 Feb 8;5(3):FSO364. doi: 10.4155/fsoa-2018-0065 (PMC6426172; doi:10.4155/fsoa-2018-0065)
Supplement: Supplementary file 3 [file fsoa-05-364-s3.docx]

# PROTOCOL FOR:

***Agrobacterium rhizogenes* mediated hairy roots transformation as a tool for exploring Aluminum responsive genes function**

**Running title: Hairy roots; tool for gene expression analysis**

**Abhijit Arun Daspute^1,2^, Xian Yunxuan^1^’^3^, Minghua Gu^3^, Yuriko Kobayashi^1^, Sopan Wagh^2^, Archana Panche^2^, Hiroyuki Koyama***

^1^Laboratory of Plant Cell Technology, Faculty of Applied Biological Sciences, Gifu University, Gifu 501–1193, Japan

^2^Institute of Bioscience and Biotechnology, MGM College, Aurangabad 411-003, India

^3^College of Agriculture, Guangxi Universities, Nanning 530-005, China

**Keywords:** *A. rhizogenes*  • *A. tumefaciens* • *AtALMT1* • Aluminum • Citrate • GFP • Malat • *STOP1* • Tobacco • *Hairy roots*

## LEGEND

🢡***ATTENTION***

* ***HINT***

**🖐*REST***

# REAGENTS AND MATERIALS

1. *A. rhizogenes* strain (ATCC^®^15438)
2. Solidified YEB medium
3. Liquid YEB medium
4. Sodium Hypochlorite (Wako, Japan)
5. 70% ethanol
6. Sterile H_2_O
7. 200 mg/ml cefotaxime stock solution (Nichi-Iko, Japan)
8. 1M acetosyringone stock solution (Sigma, USA)
9. 10 mg/ml meropenome (Wako, Japan)
10. Murashige and Skoog plant salt mixture
11. 1000x gamborg’s vitamin solution (Sigma, USA)
12. 30% glycerol
13. Sucrose

🢡***ATTENTION****: +Use all autoclaved and fully dried to avoid the contamination problem.*

**PROCEDURE**

**A.TRANSFORMATION OF AGROBACTERIUM RHIZOGENES BY ELECTROPORATION**

Modified from: Wen jun, S. and Forde, B. (1989); Efficient transformation of *Agrobacterium* spp. by high voltage electroporation. *Nucleic Acids Research* 17: 20

**a. PREPARATION OF COMPETENT CELLS**

1. *A. rhizogenes* cells grow in 100 ml of YEB broth at 28^o^C for 20-24 hrs, the final OD 600= 0.5-0.7.

2. Cool cells on ice for 20 min.

3. Transfer 40 ml of *A. rhizogenes* culture into 50 ml falcon tube.

4. Spin at 5000 g for 15 min at 4 ^o^C.

5. Wash the pellet in 40, 20, and 8 ml with chilled 10% sterile glycerol.

6. Resuspened the pellet in 1ml child 30% sterile glycerol.

7. 50μl aliquots were transferred into 1.5 ml eppendorf tubes and freeze in liquid nitrogen. Store at –80 ^o^C.

* ***HINT****: Cool the centrifuge, glycerol, falcon tubes and eppendorf tubes before starting the experiment.*

*ATTENTION: Chilled conditions can be increase transformation efficiency.*

*REST: Competent cells can be stored at -80 ^o^C upto a year.*

**b. TRANSFORMATION AND SELECTION OF DESIRED CLONE**

8. Thaw the electro competent cells on ice.

9. Add 3μl plasmid DNA (5 ng/μl) is mixed with the cell suspension and leave it for 2 min.

10. Transfer 50 μl of cells to a pre‐cooled electroporation cuvette (16 mm 125 mm) (Nepa Gene, Japan).

11. Dry the cuvette with kimwipe and an electric puls apply emediatlly using gene pulser unit (Bio Rad) by first adjusting the electroporation apparatus settings to 2.5 kV and 25 μF capacitance.

12. Immediately add 300 μl of YEB broth into the cuvette mix it gently.

13. Transfer aliquot into 1.5ml sterile eppendorf tube and shaken at 28^o^C for 3 hrs.

14. Whole aliquot is plated on YEB with agar containing appropriate antibiotics and incubated for 3 days at 28^o^C.

15. Select a colony harbouring desired construct and suspend it in 1ml YEB culture medium with appropriate antibiotic, incubate for 24 hrs at 28^o^C.

16. Pipet a 500 μl aliquot and add it in a 1.5 ml screw top tube. Add 500 μl of 30% glycerol, mix thoroughly and keep the tube at –80^o^C freezer.

**B. TRANSGENIC HAIRY ROOTS GENERATION**

Modified from: Karimi M, Van Montagu, Gheysen G. Hairy root production in Arabidopsis thaliana: cotransformation with a promoter-trap vector results in complex T-DNA integration patterns. *Plant Cell Reports* 19:133–142 (1999).

**a. SEED STERILIZATION BY USING SODIUM HYPOCHLORITE**

17. Add 100 seeds into a sterile 1.5 ml tube and add 1 ml of 1% sodium hypochlorite

18. Vertex the seed and keep it on gently mixing and leave for 5 min.

19. Speen the tube and discard the sodium hypochlorite and rinse the seeds five times with 1ml sterile water.

🢡***ATTENTION:*** *Do not incubate the seeds in sodium hypochlorite solution more than 5 min.*

🢡***ATTENTION****: Seed should be sterilised properly to avoid the contamination by microorganisms.*

**b. GERMINATION OF ARABIDOPSIS**

**🖐*REST****: Incubate the sterilized seeds in the dark at 4 ^o^C for 3 days*.

20. Place the pre incubated sterilized seeds (10 seeds of Arabidopsis per box), with the help of 10 μl pipet on the surface of solid germination medium. Seal the germination containers with surgical film and incubate for 25 days in growth camber adjusted 12 hrs light and 12 hrs darks at 21-30ºC. Seeds of god quality should give 100% germination frequency.

c. **INOCULUM PREPARATION of *A. rhizogenes*** **FOR INFECTION**

21. Streak *A. rhizogenes* from glycerol stock onto the surface of YEB plates containing the appropriate antibiotics and incubate at 28ºC for 2 days.

22. Pick a single colony and suspend in 5 ml liquid YEB medium containing appropriate antibiotics and incubate overnight at 28ºC, 160 rpm. (16-18hrs)

23. Pour the overnight grown culture into 100 ml sterile flask containing 25 ml YEB with appropriate antibiotics and 5μl Acetosyringone, incubate the culture overnight at 28ºC, 160 rpm.

24. Pellet cells at 3,000 rpm, 15 min at room temperature (24^o^C), remove supernatant, add 20 ml of ¼ MS with 1% sucrose media and resuspend.

25. Repeat Step 24 twice.

🢡***ATTENTION:*** *These washes remove traces of antibiotic, which maybe kill the leaf tissue after infection.*

**d. INDUCTION OF HAIRY ROOTS TIMING**

26. Cut the ex-plants (Arabidopsis, steam; tobacco, leaf) with a sterile scalpel /scissor, and placed in petri plate containing 20 ml ¼ MS liquid + 1% sucrose solution. Further cut can be made in MS.

🢡***ATTENTION:*** *Leaf should be cut transversely and care should be taken with the forceps so that it don’t cause excessive wounding to the ex-plant.*

🢡***ATTENTION:*** *Do not use plants with low vigour; this influences the formation of hairy roots.*

27. Transfer the explants into 50 ml tube containing diluted *A. rhizogenes* and soak for 20 min.

🢡***ATTENTION:*** *Do not soak ex-plants long time in the A. rhizogenes solution; this may negatively influence the hairy roots formation.*

28. Remove the bacterial solution and place the ex-plants on sterile paper towel, allow to blot excess bacteria.

🢡***ATTENTION:*** *Poor blotting may led to kill the ex-plants during co-cultivation.*

29. Transfer explants onto solid MS plates (1% sucrose, acetosyringone, no antibiotics), 10 explants per plate (abaxial side up in case of leaf as a explants) and 20 steam explants.

30. Seal the plates with surgical film and Co-cultivate for 3 days in dark at 22 to 25^o^C.

**🖐*REST****: Co-cultivation can be done for 3 days.*

31. After 3 days, transfer the 5 leaf and 10 steam ex-plants onto root selection medium, abaxial side up (leaf ex-plants); lay the explants as flat as possible on medium by using the back of forceps, emphasizing contact of the wounded end onto the medium.

32. Seal the plats and incubate at 22-25^o^C until hairy roots grows.

33. Once hairy roots are at least 1.0 cm long, roots can be excised from the explants and transfer individual root to root selection medium.

34. Antibiotic concentration can be reduced after 3-4 round of hairy roots selection

35. The well developed hairy roots can be directly used for functional genomics studies or maintained in liquid MS medium under antibiotic selection or can be maintained on solid MS medium for further use.

**REAGENT SETUP**

YEB medium

Yeast extract (1.0 g/l), Nutrient broth (5.0 g/l), Tryptone (5.0 g/l), Sucrose (5.0 g/l), MgSO_4_ _ 7H_2_O (0.5 g/l), pH 7.0. Also include the proper antibiotic resistance selection drug for the GFP/GUS fusion construct carried by these bacteria.

Solid germination medium for plant growth

(Add 2.15 g/l MS, 0.8% agar and 1.0% sucrose, Dissolve Murashige and Skoog salts and sucrose in ultrapure deionized water. Before adding the agar, adjust the pH to 5.8, autoclave the meadiam for 20 min. at121^o^C. When the medium get cooled to 50 ^o^C, pour into petri dishes or glass boatels.

Solid co-cultivation medium

(Add 2.15 g/l MS, 1.2 % agar, 1.0% sucrose and 200 μl/l acetosyringone (Sigma, USA, 1M stock). Dissolve Murashige and Skoog salts and sucrose in ultrapure deionized water. Adjust the pH to 5.8 and autoclave. When the medium get cooled to 50 ^o^C, add filter-sterilized acetosyringone and pour into Petri dishes.

Selection and root induction medium

(Add 2.15 g/l MS, 1.0 % agar, 1.0% sucrose, 200 mg/L cefotaxime sulphate (Nichi-Iko, Japan; 200 mg /ml stock), 6.25 mg /l meropenome (Wako, Japan; 10 mg/ ml stock), 1x gamborg vitamin (Sigma, USA; 1,000x stock) and 1000 μl/l kanamycine ( 50 mg/ml stock). Dissolve Murashige and Skoog salts and sucrose in ultrapure deionized water. Adjust the pH to 5.8 and autoclave. When the medium has cooled to 50^o^C, add filter-sterilized antibiotics and pour into petri dishes.

🢡***ATTENTION:*** *These additions are heat labile and therefore should only be added when the medium is get cool. Poured medium plates are kept at 4 ^o^C in the dark.*

**TROUBLESHOOTING**

POOR GERMINATION

-The seeds were incubated too long in sodium hypochlorite solution. Do not incubate seed more than 5 min. in sodium hypochlorite solution.

-The seeds were not rinsed properly after sodium hypochlorite solution treatment. Rinse the seeds at least 5 times by using distilled water.

AGROBACTERIUM RHIZOGENES INOCULUMS CONTAMINATION

-Use autoclaved test tubes and conical flasks.

-Add appropriate antibiotics.

EX-PANTS DEAD DURING COCULTIVATION

-Wash inoculums using ¼ MS at least 2 times.

-Properly blot the excess bacteria.

LACK OF ROOTS FORMATION

-The ex-plants are too old, use young seedlings (28 days old Arabidopsis and 15 days old tobacco).

-Antibiotic pressure is too high. Optimize the antibiotic concentration.

EXCESS ROOT FORMATION WITH MAJORITY OF NON TRANSFORMANTS

-Antibiotic concentration is too less. Optimize the antibiotic concentration.
